# Supplementary material for: The intestinal clock drives the microbiome to maintain gastrointestinal homeostasis
Source: Nat Commun. 2022 Oct 14;13:6068. doi: 10.1038/s41467-022-33609-x (PMC9568547; doi:10.1038/s41467-022-33609-x)
Supplement: Supplementary file 1 — Supplementary Information [file 41467_2022_33609_MOESM1_ESM.pdf]

Supplementary Information

The Intestinal Circadian Clock Drives the Microbiome to Maintain Gastrointestinal Homeostasis

Marjolein Heddes[1, 2,†] , Baraa Altaha[1, 2,†], Yunhui Niu[1, 2], Sandra Reitmeier[1, 2], Karin Kleigrewe[3], Dirk Haller[1, 2,], Silke Kiessling [1,2,4,*]

*Corresponding Author: Dr. Silke Kiessling, silke.kiessling@tum.de

[†]These authors contributed equally

[1] ZIEL - Institute for Food & Health, Technical University of Munich, 85354 Freising, Germany

[2] Chair of Nutrition and Immunology, Technical University of Munich, Gregor-Mendel-Str. 2, 85354 Freising, Germany

[3] Bavarian Center for Biomolecular Mass Spectrometry, Technical University of Munich, Gregor-Mendel-Str. 4, 85354 Freising, Germany

[4] Faculty of Health and Biomedical Science, University of Surrey, Stagg Hill Campus, GU27XH, Guildford, UK

Table of Contents

Supplementary Figures

Suppl. Figure 1: Diurnal and Circadian Rhythms in Behavior and Microbiota Composition

Suppl. Figure 2: Characterization of Rhythmic Behavior and Microbial Profiling of Bmal1$^{IEC-/-}$ Mice

Suppl. Figure 3: Bmal1$^{IEC-/-}$ and Control Microbial transfer to Germ-free Mice

Suppl. Figure 4: Loss of Cecal Microbial Oscillations and Function in Bmal1$^{IEC-/-}$ Mice.

Suppl. Figure 5: Immune Cell Recruitment and Gene Expression in SPF Donors and Germ-free Mice after Microbiota Transfer

Suppl. Figure 6: Top 10 Differently Regulated Predicted Microbial Pathways in Recipients

Suppl. Figure 7: FACS Gating Strategy

Supplementary Figure 1

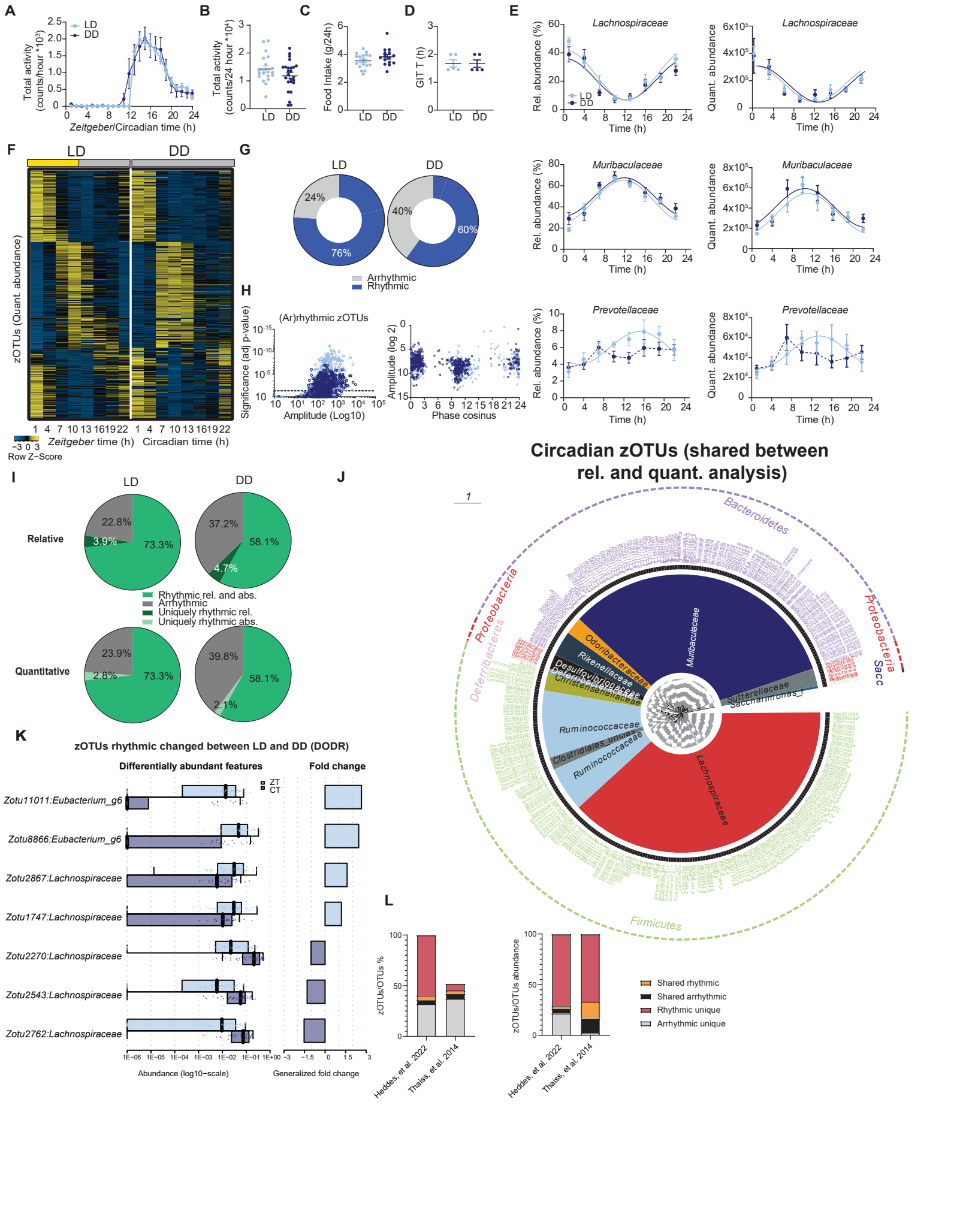

Supplementary Figure 1. Diurnal and Circadian Rhythms in Behavior and Microbiota Composition
(A) Diurnal (LD) and circadian (DD) total wheel-running activity profiles and 24-h summary (B) n=20(LD);25(DD). Total daily food intake (n=15(DD);17(LD)) (C) and gastro-intestinal transit time (GITT) (n = 5/condition) (D). (E) Diurnal and circadian profile of relative (left) and quantitative (right) abundance of the major family of fecal micro-biota. (F) Heatmap depicting the quantitative abundance of 580 zOTUs (mean relative abundance > 0.1%; prevalence > 10%). Data are normalized to the peak of each zOTU and ordered by the peak phase in LD conditions. (G) Pie-charts indicate the amount of rhythmic (blue) and arrhythmic (grey) zOTUs, identified as rhythmic (Bonferroni adj. p-value ≤ 0.05) by JTK_Cycle. (H) Significance (Bonferroni adj. p-value based on JTK_Cycle) and amplitude of rhythmic and arrhythmic zOTUs (left) and phase distribution (right) in LD and DD based on quantitative analysis. Dashed line indicates p-value = 0.05. (I) Pie charts indicating percentage of overlap in rhythmic (green) and arrhythmic (grey) zOTUs between relative (top) and quantitative (bottom) analyses in LD (left) and DD (right) conditions, identified as rhythmic (Bonferroni adj. p-value ≤ 0.05) by JTK_Cycle. (J) Taxonomic tree of circadian zOTUs shared by both relative and quanti-tative analyses. Taxonomic ranks are from phylum (outer dashed ring), family (inner ring) to genera (middle, color coded according to phylum) indicat-ed by individual branches. (K) Box and bar plots illustrate the alteration in relative abundance and fold change between LD and DD of zOTUs (two-sided Wilcoxon, adj. p-value ≤ 0.05), which showed altered rhythmicity according to the adjusted compare rhythm script based on DODR. (L) Bar charts comparing rhythmic/arrhythmic zOTUs/OTUs percentage (left) and abundance (right) of Thaiss et al. and Heddes et al. Data were normalized to the amount of zOTUs for percentage calculation, identified as rhythmic (Bonferroni adj. p-value ≤ 0.05) by JTK_Cycle. Significant rhythms are illustrated with fitted cosine-re-gression solid line; data points connected by dotted lines indicate no significant cosine fit curve (p-value > 0.05) and thus no rhythmicity. LD (light-blue) and DD (dark-blue). n = 6 mice/time point/light condition unless otherwise indicated. Data are represented as mean ± SEM. Source data are provided as a source data file.

Supplementary Figure 2

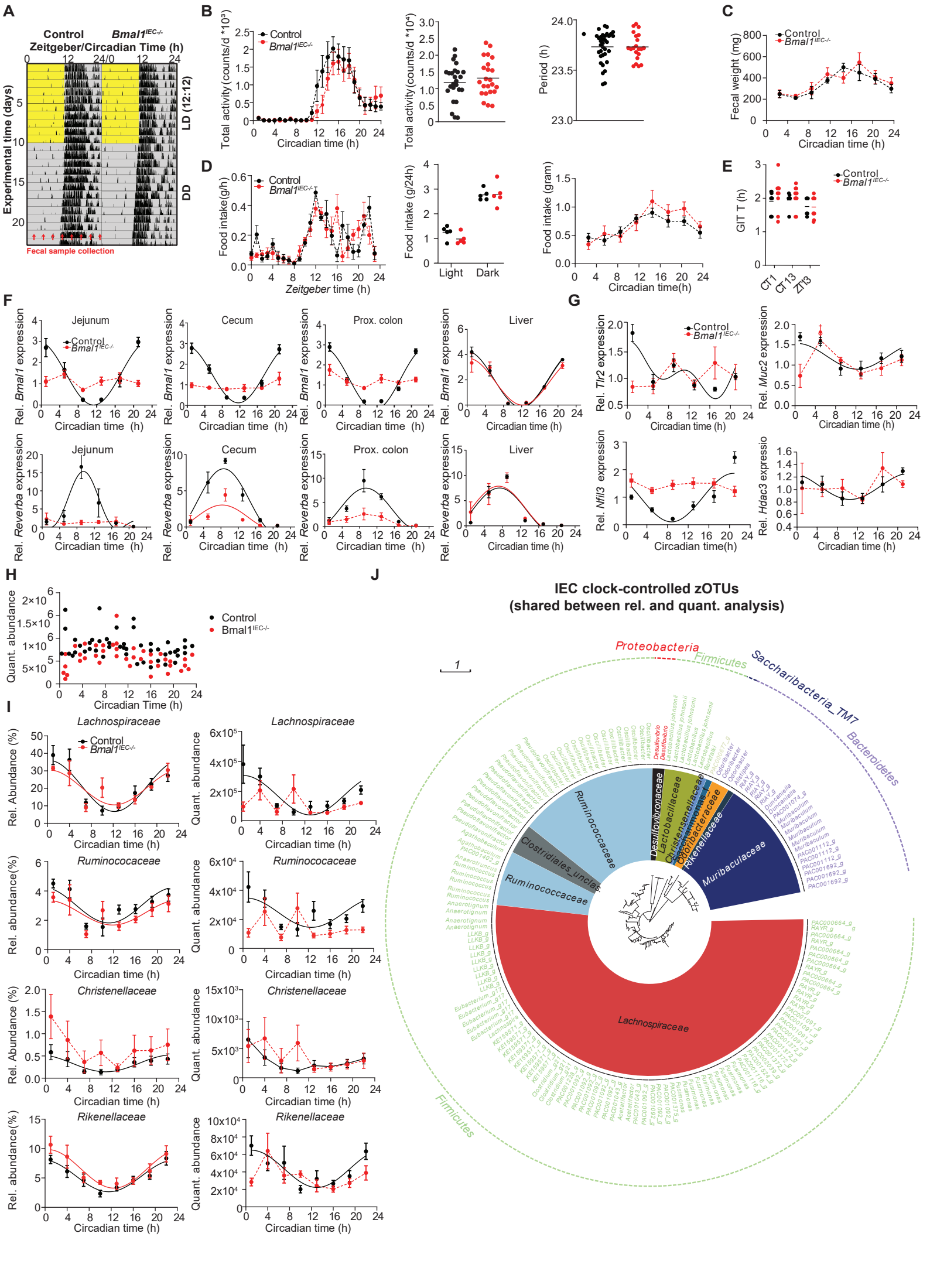

Supplementary Figure 2. Characterization of Rhythmic Behavior and Microbial Profiling of *Bmal1*$^{IEC-/-}$ mice.
(A) Representative actogram in LD and DD conditions of *Bmal1*$^{IECfl/fl}$ controls and *Bmal1*$^{IEC-/-}$ mice, red arrows indicate fecal samples collection time points. (B) Activity profile of *Bmal1*$^{IEC-/-}$ (n = 12) and control mice (n = 11) in light-dark (LD) cycle (left) and the quantification of circadian activity (middle, control n=25, Bmal1$^{IEC-/-}$ n=23) and the period in DD (right, control n=25, Bmal1$^{IEC-/-}$ n=18) as well as (C) fecal weight in DD over time (control n=8, Bmal1$^{IEC-/-}$ n=7) and (D) food intake diurnal profile and its average (middle, (n=5/genotype), and food intake circadian profile (right, control n=8, Bmal1$^{IEC-/-}$ n=7) (E) GITT in LD and DD (n = 6(control); 8(*Bmal1*$^{IEC-/-}$)). Expression profiles of core clock genes (n=4/genotype) (F) and clock-controlled genes (G) (n= 24 control and 25 *Bmal1*$^{IEC-/-}$ mice, repeated measures) . (H) 16S copy number over time (2-way ANOVA) (I) Circadian profiles at family level of relative abundance (left) and quantitative abundance (right) of control (n=6) and *Bmal1*$^{IEC-/-}$ (n = 5) mice. (J) Taxonomic tree of fecal circadian gut clock controlled microbiota uniquely rhythmic in control mice in both relative and quantitative analyses. Taxonomic ranks are from phylum (outer dashed ring), family (inner ring highlighted) to genera (middle, color coded according to phylum) which are indicated by the individual branches. Significant rhyhms are illustrated with fitted cosine-regression or fitted harmonic-regression; data points connected by dotted lines indicate no significant cosine fit curves (p-value > 0.05) and thus no rhythmicity. *Bmal1*$^{IEC-/-}$ (red) and control (black). Data are represented as mean ± SEM. Significance: p-value ≤ 0.05. Source data are provided as a source data file.

Supplemantary Figure 3

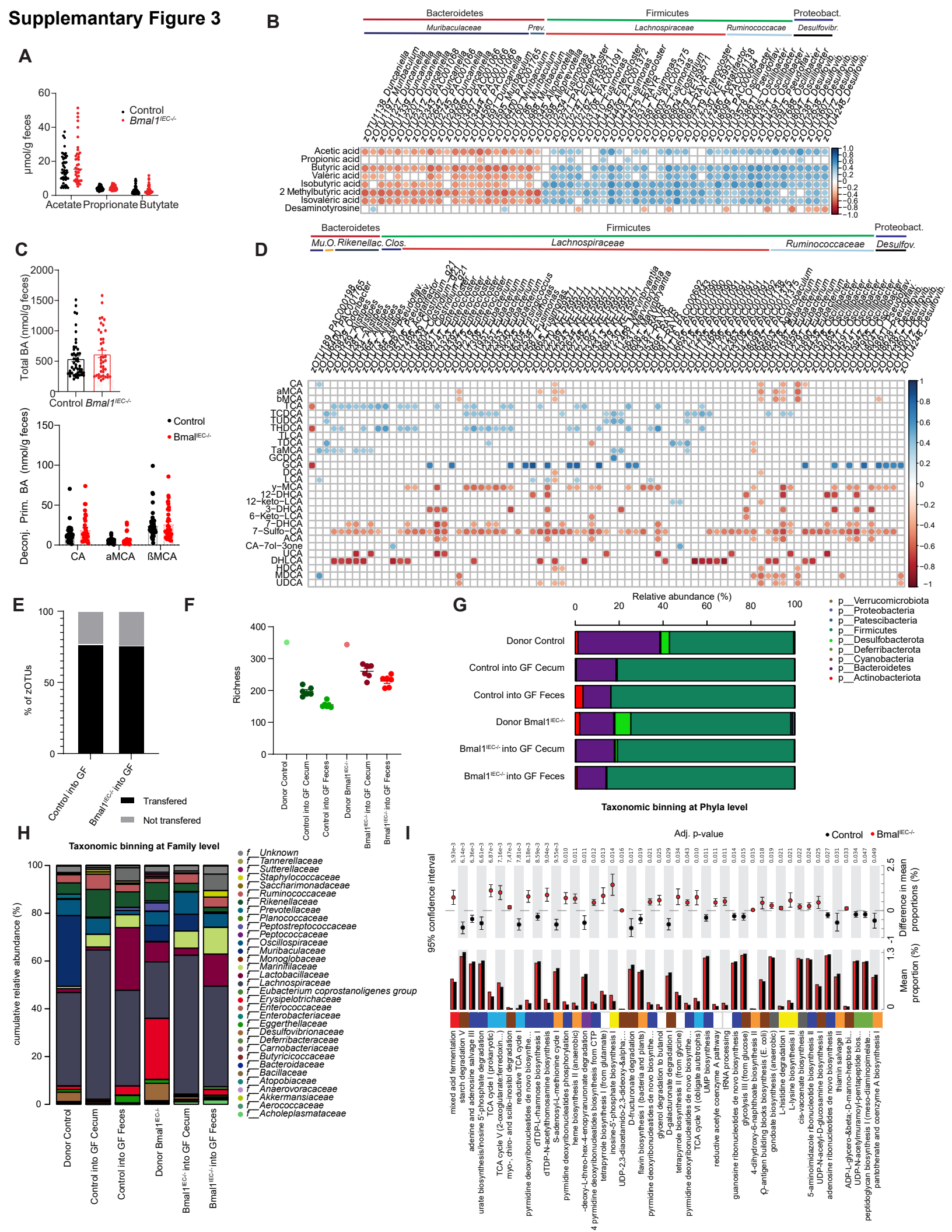

Supplementary Figure 3. Arrhythmic microbial transfer to germ-free mice.
(A) Total SCFA concentrations in feces n=6(control)/5(Bmal1$^{IEC-/-}$)mice/time point/genotype (repeated measures). (B) Spearman correlation of SCFA (p-value ≤ 0.05 and R ≤ -0.5 (red) or R ≥ 0.5 (blue)) with gut-controlled bacteria taxa. Legend indicates the correlation coeffcieint (R) with red representing a negative correlation and blue representing a positive correlation (C) total and deconjugated bile-acid levels in feces (n=6(control)/5(Bmal1$^{IEC-/-}$)mice/time point/genotype (repeated measures)) and their (D) Spearman correlation of bile-acids (p-value ≤ 0.05 and R ≤ -0.5 (red) or R ≥ 0.5 (blue)) with gut-controlled bacteria taxa. n=6(control)/5(Bmal1$^{IEC-/-}$)mice/time point/genotype (repeated measures). Legend indicates the correlation coeffcient (R) with red representing a negative correlation and blue representing a positive correlation. (E) Percentage of zOTUs transferred (grey) into recipient mice (n = 6/geno-type). (F) Richness of donor (n = 4 mixture) and recipient samples collected at CT13/ZT13. Taxonomic binding of microbiota from donor and recipient mice at CT13/ZT13 at phyla (G) and family (H) level. (I) Pathways predicted using PICRUST2.0 on intestine clock-controlled zOTUs showing significant differences in abundance between genotypes n=6 mice/time point/genotype. Pathways are colored according to their sub-class. Statistical differences for Picrust data were calculated based on White's non-parametric two-sided t-test and Benjamini Hochberg dales discovery rate to adjusted for multiple testing. All data are presented as mean values +/- SEM. Control (black) and Bmal1IEC-/- (red). Cholic acid (CA), a-Murichолic acid (aMCA), b-Muricholic acid (bMCA), Taurocholic acid (TCA), Taurochenodeoxycholic acid (TCDCA), Tauroursodeoxycholic acid (TUDCA), Taurohyodeoxycholic acid (THDCA), Taurolithocholic acid (TLCA), Taurodeoxycholic acid (TDCA), Tauro-a-Muricholic acid (TaMCA), Glycochenodeoxycholic acid (GCDCA), Glycocholic acid (GCA), Deoxycholic acid (DCA), Lithocholic acid (LCA), y-Muricholic acid (y-MCA), 12-De-hydrocholic acid (12-DHCA), 12-Ketolithocholic acid (12-keto-LCA), 3-Dehydrocholic acid (3-DHCA), 6-Ketolithocholic acid (6-keto-LCA), 7-Dehy-drocholic acid (7-DHCA), 7-Sulfocholic acid (7-sulfo-CA), Allocholic acid (ACA), Cholic-acid-7ol-3one (CA-7ol-3one), Ursocholic acid (UCA), Dehy-drolithocholic acid (DHLCA), Hyodeoxycholic acid (HDCA), Murideoxycholic acid (MDCA), Ursodeoxycholic acid (UDCA). Source data are provided as a source data file.

Supplementary Figure 4

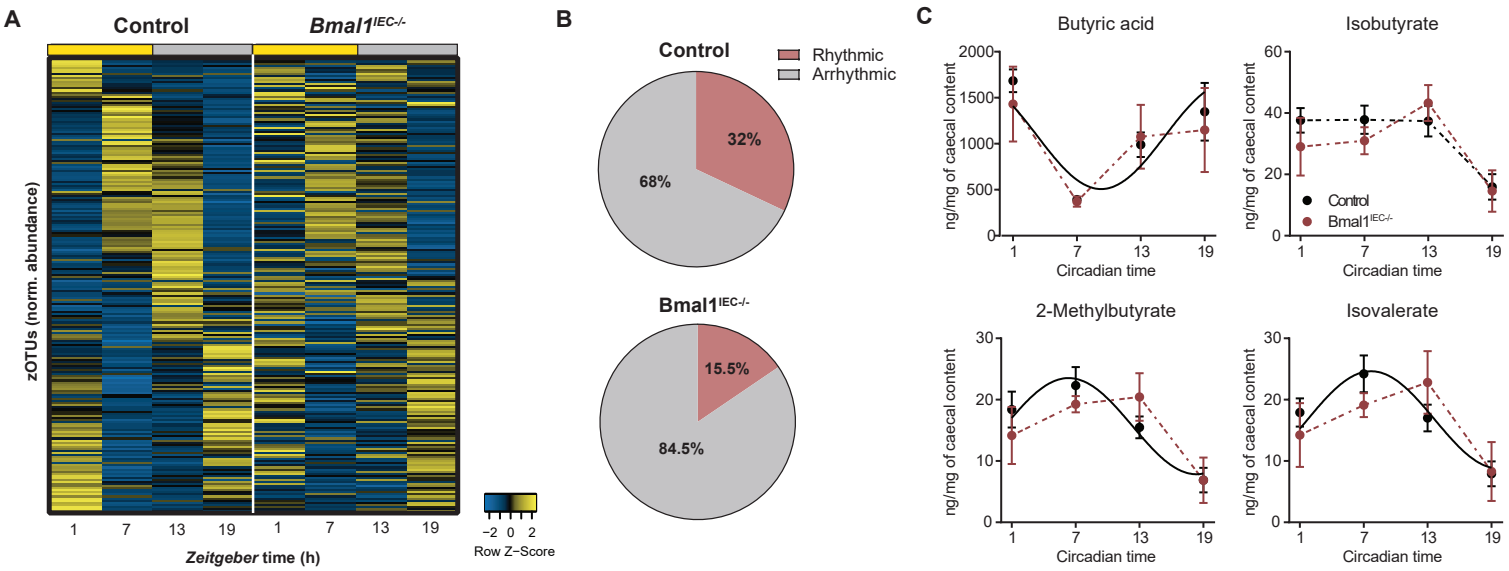

Supplementary Figure 4. Loss of cecal microbial oscillations and function in Bmal1$^{IEC-/-}$ mice.

(A) Heatmap depicting zOTUs over time in control (left) and Bmal1IEC-/- mice (right). (B) Pie-charts indicating total percentage of rhythmic (green) and arrhythmic (grey) zOTUs). (C) Circadian profiles of cecal SCFAs. n=14 Bmal1IEC-/- (repeated measures); n=21 control (repeated measures). Significant rhythms (Bonferroni adj. p-value <0.05, identified by JTK_cycle) are illustrated with fitted cosine-regression (solid line); data points connected by dotted lines indicate no significant cosine fit curves (Bonferroni adj. p-value > 0.05 based on JTK_cycle) and thus no rhythmicity. Control (black) and Bmal1IEC-/- (red-brown). Data are represented as mean ± SEM. Source data are provided as a source data file.

Supplementary Figure 5

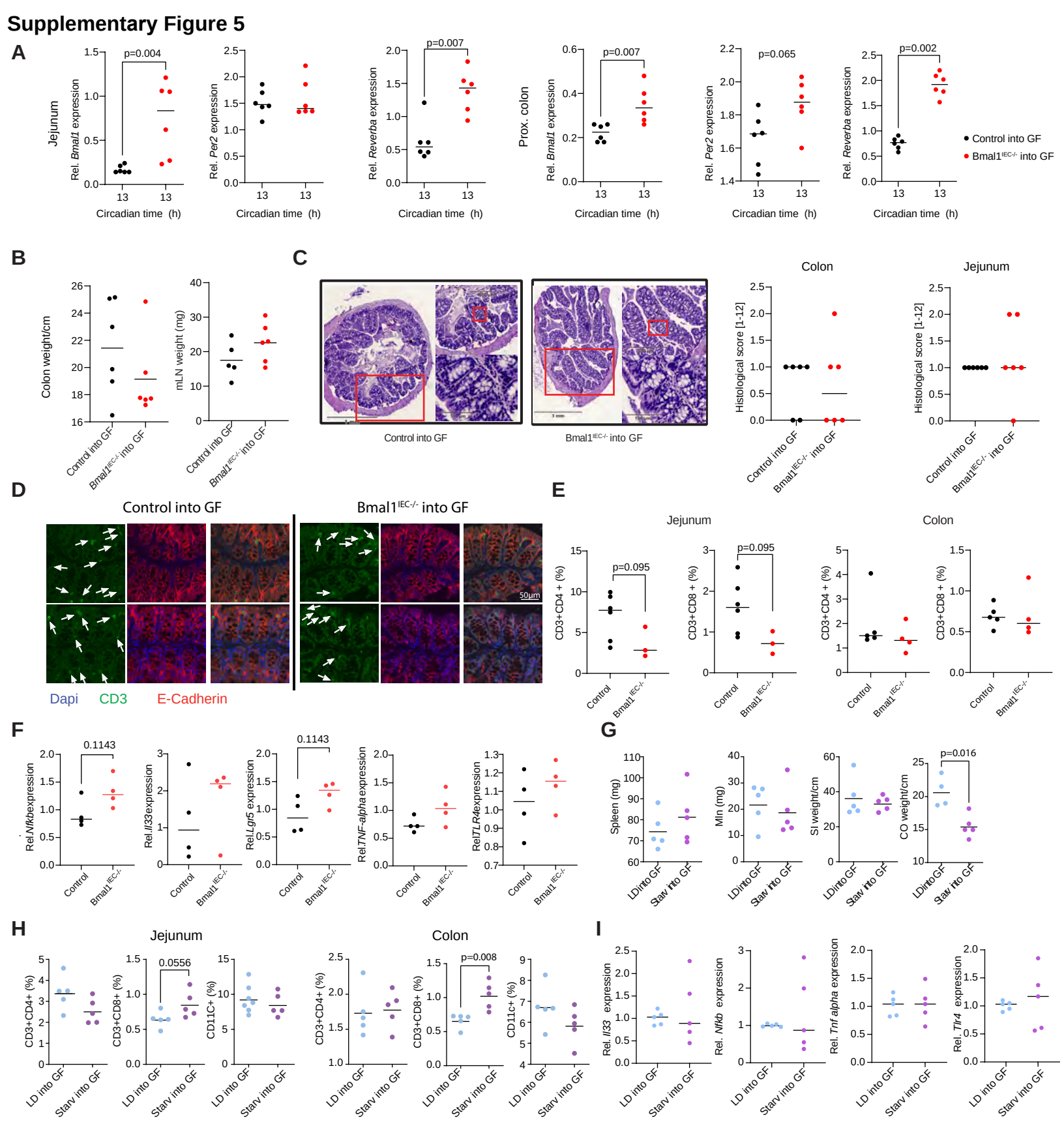

Supplementary Figure 5. Immune cell recruitment and gene expression in SPF donors and germ free mice after microbiota transfer. (A) Clock gene expression measured at CT13 in Jejunum (left) and proximal colon (right) of recipient mice 6 weeks after microbiota transfer of *Bmal1*$^{IECfl/fl}$ control (black) or *Bmal1*$^{IEC-/-}$ mice (red). (B) Organ weights of recipient mice after receiving control or *Bmal1*$^{IEC-/-}$ cecal microbiota. (C) Cross section of proximal colon along with the histological scoring of proximal colon and jejunum of germ-free mice after receiving control or *Bmal1*$^{IEC-/-}$ cecal microbiota. (D) Immunofluorescence staining of CD3 (green), Ecadherin (red) and Dapi (blue) of proximal colon of germ-free mice after receiving control or *Bmal1*$^{IEC-/-}$ cecal microbiota (pictures are from representative samples of n =3/group staining repeated at least twice. (E) Frequency of CD3+CD4+, CD3+CD8+ cells in jejunum (n=6(control); n=3(*Bmal1* $^{IEC-/-}$)) and colon (n=5(control;n=4(*Bmal1*$^{IEC-/-}$)) of SPF (donor) mice. (F) Relative gene expression of Tlr4, Tnfa, Il33, Nfkb, Lgr5 in the proximal colon of SPF control and *Bmal1*$^{IEC-/-}$ mice (n = 4/geno-type).(G) Organ weights of recipient mice after receiving LD (blue) or starvation (purple) microbiota. (H) Frequency of CD3+CD4+, CD3+CD8+ and CD11c+ cells in jejunum and colon after transfer of LD microbiota and starvation microbiota into GF-BL6 recipients. (I) Relative gene expression of Tlr4, Tnfa, Il33, Nfkb, Ang4 in the proximal colon into LD microbiota and starvation microbiota recipient mice. Data are represented as mean ± SEM. * p ≤ 0.05, ** p ≤ 0.01 (Mann-Whitney U test, two-sided). n=6/genotype (a,b,c); n=5/genotype(g,h,i). Source data are provided as a source data file.

Supplementary Figure 6

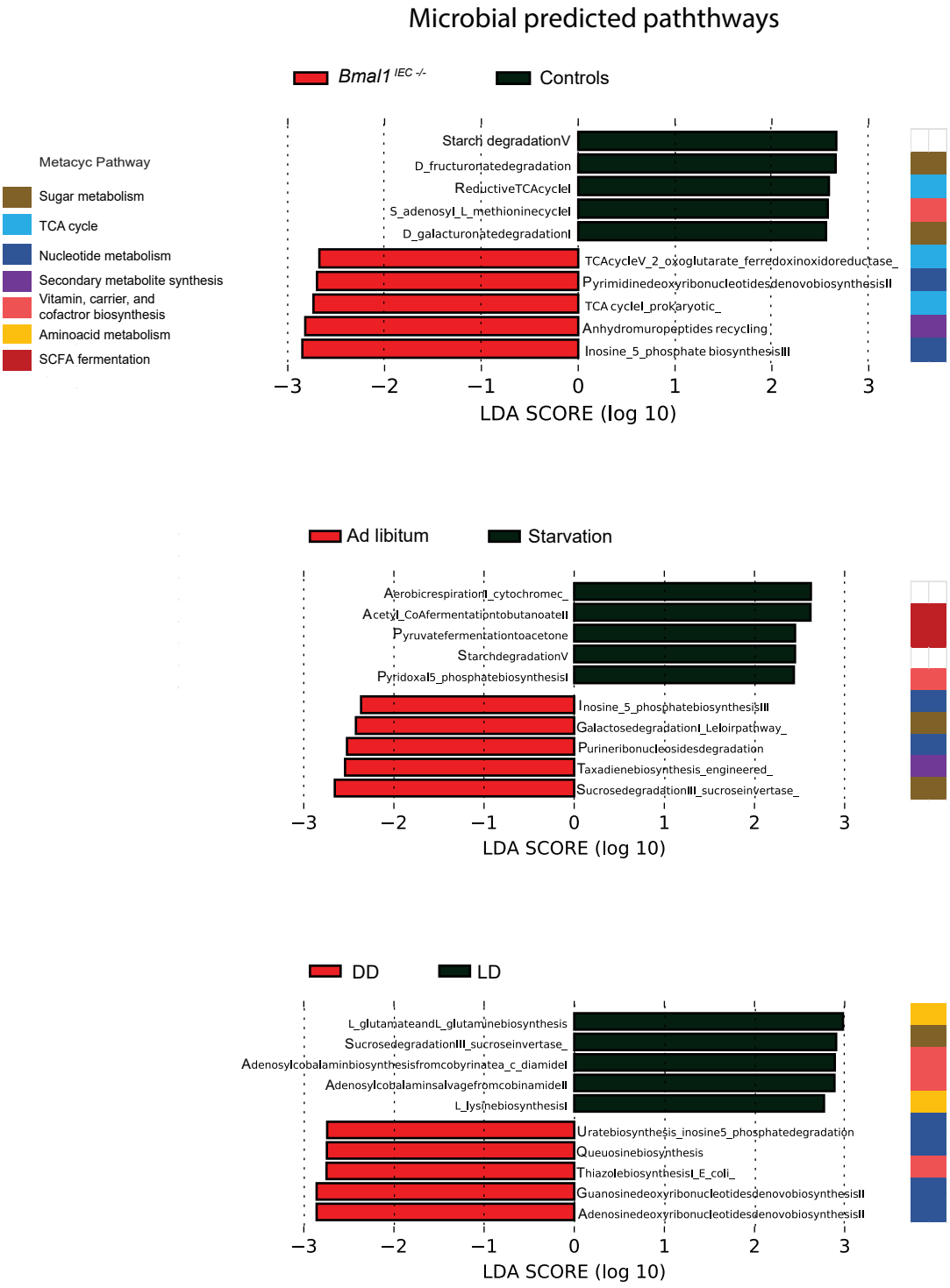

Supplementary Figure 6. Top 10 Differently Regulated Predicted Microbial Pathways.
Pathways altered in gut clock deficient mice (Bmal1IEC-/-, top), food deprivation (middle), and constant darkness (DD) (bottom), in comparisons to their controls. Microbial pathways were assessed based on PICRUST 2.0 and were compared based on LDA score. n=48 Bmal1IEC-/-/Control; n=83 ad libitum; n=81 starvation; n=48 DD/LD. Source data are provided as a source data file.

Supplementary Figure 7

A

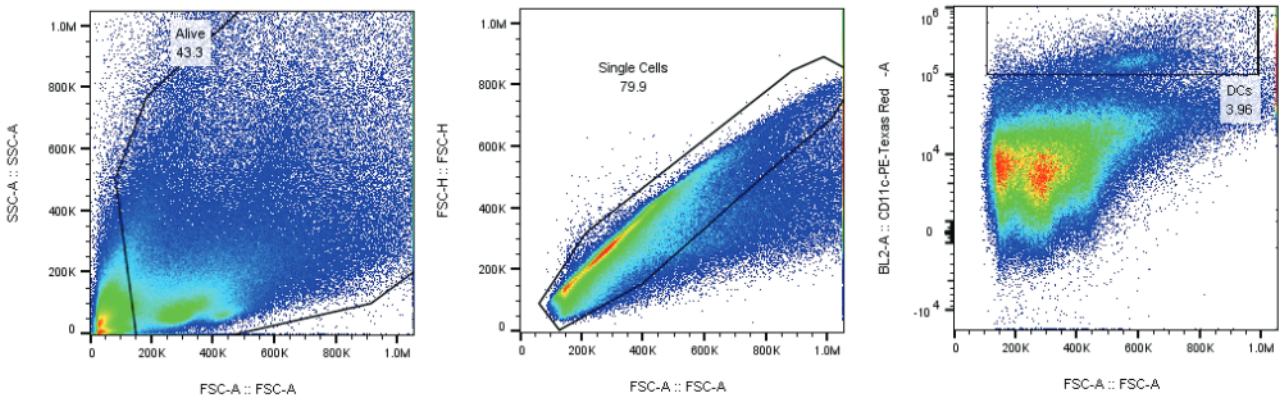

B

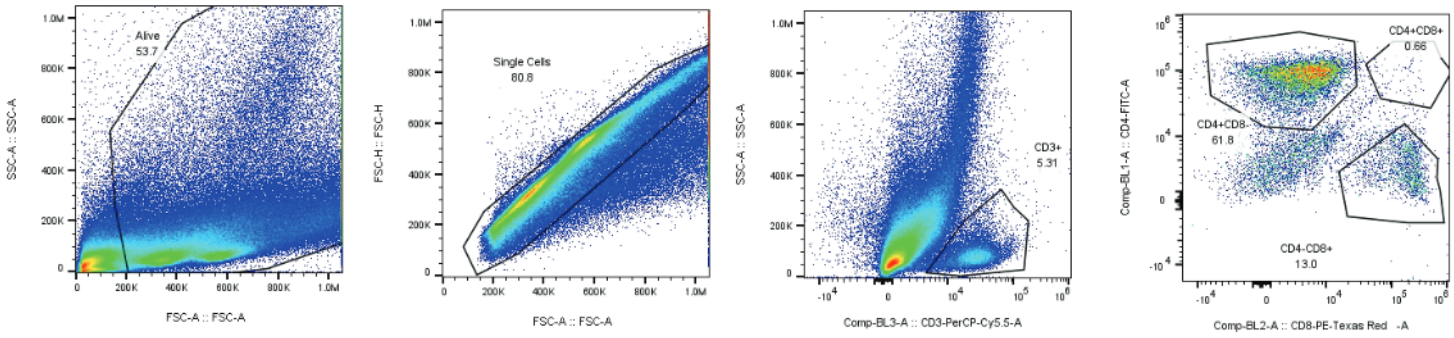

Supplementary Figure 7. FACS gating strategy.
Gating strategy of DCs (A) and CD3+CD4+, CD3+CD8+ (B). Plots are shown from a representative sample. The numbers in the plots indicate percentage of cells within each gate.
