# Supplementary material for: The intestinal clock drives the microbiome to maintain gastrointestinal homeostasis
Source: Nat Commun. 2022 Oct 14;13:6068. doi: 10.1038/s41467-022-33609-x (PMC9568547; doi:10.1038/s41467-022-33609-x)
Supplement: Supplementary file 2 — Description of Additional Supplementary Files [file 41467_2022_33609_MOESM2_ESM.pdf]

Description of Additional Supplementary Files

File name: **Supplementary Data 1**
Description: Rhythmicity and amplitude analysis of zOTUs using JTK_cycle, as well as the average abundance and phase of each zOTU.

File name: **Supplementary Data 2**
Description: Percentage of rhythmic/arrhythmic zOTUs that belongs to Firmicutes, Bacteriodetes or other phyla.

File name: **Supplementary Data 3**
Description: Compare rhythm analysis of absolute abundance of zOTUs.

File name: **Supplementary Data 4**
Description: comparison of rhythmic/ arrhythmic zOTUs from Heddes et al. LD cohort in comparison to Thaiss et al. 2014 data.

File name: **Supplementary Data 5**
Description: p-value of amplitude, baseline and phase shift comparisons of all 24-hour circadian profiles provided in the figures.
