# Supplementary material for: The intestinal clock drives the microbiome to maintain gastrointestinal homeostasis
Source: Nat Commun. 2022 Oct 14;13:6068. doi: 10.1038/s41467-022-33609-x (PMC9568547; doi:10.1038/s41467-022-33609-x)
Supplement: Supplementary file 8 — Reporting Summary [file 41467_2022_33609_MOESM8_ESM.pdf]

nature portfolio

Corresponding author(s): Dr. Silke Kiessling

Last updated by author(s): 7/9/2022

Reporting Summary

Nature Portfolio wishes to improve the reproducibility of the work that we publish. This form provides structure for consistency and transparency in reporting. For further information on Nature Portfolio policies, see our Editorial Policies and the Editorial Policy Checklist.

Statistics

For all statistical analyses, confirm that the following items are present in the figure legend, table legend, main text, or Methods section.

n/a	Confirmed	
☐	☒	The exact sample size (*n*) for each experimental group/condition, given as a discrete number and unit of measurement
☐	☒	A statement on whether measurements were taken from distinct samples or whether the same sample was measured repeatedly
☐	☒	The statistical test(s) used AND whether they are one- or two-sided<br>*Only common tests should be described solely by name; describe more complex techniques in the Methods section.*
☐	☒	A description of all covariates tested
☐	☒	A description of any assumptions or corrections, such as tests of normality and adjustment for multiple comparisons
☐	☒	A full description of the statistical parameters including central tendency (e.g. means) or other basic estimates (e.g. regression coefficient) AND variation (e.g. standard deviation) or associated estimates of uncertainty (e.g. confidence intervals)
☐	☒	For null hypothesis testing, the test statistic (e.g. *F*, *t*, *r*) with confidence intervals, effect sizes, degrees of freedom and *P* value noted<br>*Give P values as exact values whenever suitable.*
☒	☐	For Bayesian analysis, information on the choice of priors and Markov chain Monte Carlo settings
☒	☐	For hierarchical and complex designs, identification of the appropriate level for tests and full reporting of outcomes
☐	☒	Estimates of effect sizes (e.g. Cohen's *d*, Pearson's *r*), indicating how they were calculated

Our web collection on statistics for biologists contains articles on many of the points above.

Software and code

Policy information about availability of computer code

Data collection	No software was used
Data analysis	GraphPad Prism, version 9.0.0, heatmapper.ca, R package "corrplot" v0.92, R package SIAMCAT v1.10.0, R package JTK_cycle v3.1, STAMP (2.1.3), FlowJo software (10.7.2), ClockLab software v6.0.52 (Actimetrics). Adjusted version of Compare rhythm R package (DOI: 10.5281/zenodo.7049842). Rhea pipeline R package (v1.1.5).

For manuscripts utilizing custom algorithms or software that are central to the research but not yet described in published literature, software must be made available to editors and reviewers. We strongly encourage code deposition in a community repository (e.g. GitHub). See the Nature Portfolio guidelines for submitting code & software for further information.

Data

Policy information about availability of data

All manuscripts must include a data availability statement. This statement should provide the following information, where applicable:

- Accession codes, unique identifiers, or web links for publicly available datasets
- A description of any restrictions on data availability
- For clinical datasets or third party data, please ensure that the statement adheres to our policy

Metabolite dataset can be found on Zenodo: 10.5281/zenodo.6962318 and 16s rRNA sequencing data set can be found on the SRA data bank from NCBI with submission ID: SUB10646422/Bioproject ID: PRJNA779969

Human research participants

Policy information about studies involving human research participants and Sex and Gender in Research.

Reporting on sex and gender	not applicable
Population characteristics	not applicable
Recruitment	not applicable
Ethics oversight	not applicable

Note that full information on the approval of the study protocol must also be provided in the manuscript.

Field-specific reporting

Please select the one below that is the best fit for your research. If you are not sure, read the appropriate sections before making your selection.

☒ Life sciences ☐ Behavioural & social sciences ☐ Ecological, evolutionary & environmental sciences

For a reference copy of the document with all sections, see nature.com/documents/nr-reporting-summary-flat.pdf

Life sciences study design

All studies must disclose on these points even when the disclosure is negative.

Sample size	Most publications made use o only 2 time points (12 h apart) and call thier results "circadian". However, we aim to always provide the highest resolution possible (i.e. as many time points as possible over 24 h). Although a minimum of 4 time points (i.e. every 6 h) and 3 samples per time point is a rather common procedure in the circadian field, in our study we used 6 time points and 4-6 samples per time point, being highly above average of what is mostly published. In all of our experiments, given the high amplitude of the rhythms or the large differences between groups, the group size appear to be large enough to provide sufficient power to yield statistically significant differences. This has been confirmed by Portaluppi et al., where they recommend for animal studies to use n > 3 every 4 h during a 24-h cycle, for proper assessment of biological rhythms (Portaluppi, Francesco et al., (2010). ETHICS AND METHODS FOR BIOLOGICAL RHYTHM RESEARCH ON ANIMALS AND HUMAN BEINGS. Chronobiology International, 27(9-10), 1911–1929).
Data exclusions	2x standard deviation outlier rule was used in very few exceptions
Replication	Fecal 16s sequencing regarding the control mice and Bmal1IEC-/- mice have been performed 3x with similar results. All animal experiements presented in the paper were repeated in multiple animals (n=3-6/experiment) and findings were reproducible. Microbial analyses and profiling were performed on all mice included in the study and experimental findings in mice were reproducible. Tissue analysis, imaging and immune phenotyping, gene expression profiles as well as activity data are all based on multiple animals for a given experiment.
Randomization	For almost all experiments, different experimental conditions littermates/genotypes were randomly divided into the experimental groups with the use of excel. One exception are the germ-free experiments, mice were not randomized into the experimental transfer groups. Due to the capacity of the isolators, only a set of 12 mice and one microbial gavage could be performed per experiment.
Blinding	Histological analyses was performed double blinded by an independent scientist. FACS quantifications , CD3 immunofluorescence staining analyses as well as activity analyses were performed blinded. Other experiments were not performed blinded, as mice were assigned to certain treatments e.g. starvation/microbial gavage and researchers knew which mouse received which treatment.

Reporting for specific materials, systems and methods

We require information from authors about some types of materials, experimental systems and methods used in many studies. Here, indicate whether each material, system or method listed is relevant to your study. If you are not sure if a list item applies to your research, read the appropriate section before selecting a response.

Materials & experimental systems

n/a	Involved in the study	
	x	Antibodies
x		Eukaryotic cell lines
x		Palaeontology and archaeology
	x	Animals and other organisms
x		Clinical data
x		Dual use research of concern

Methods

n/a	Involved in the study	
x		ChIP-seq
	x	Flow cytometry
x		MRI-based neuroimaging

Antibodies

Antibodies used

Immuno flurescence
- Anti E-cadherin (mouse), Lot GR3360021-1, CAT ab76055 ,clone M168 (Abcam)
- Anti-CD3 (rabbit) C7930, Lot 065M4814V (Sigma)
- Secondary antibodies;
donkey anti rabbit 546 (Invitrogen), Lot 1833519, Ref A10040
donkey anti mouse 647 (Invitrogen), Lot 2260928, Ref A31571

And for FACS:
- PE Rat Anti-Mouse CD8a clone 53-6.7, lot 0322142, CAT 553033 (BD)
- PerCP/Cyanine5.5 anti mouse CD3 clone 17A2, lot B314600, CAT 100218 (Biolegend)
- PE/Cyanine7 anti mouse IL-17A clone TC11-18Hl0.l, lot B303324, CAT 506922 (Biolegend)
- FITC Rat Anti-mouse CD4 Clone RM4-5, Lot 0076381, CAT 553046 (BD)
- APC Anti-Mo IFN gamma, Clone XMG 1.2 Lot 2175632, Ref 17-7311-82 (Invitrogen)
- PE Hamster Anti-mouse CD11c, clone HL3, LOT 7096716, CAT 553802 (BD)
- APC/Cyanine7 anti-mouse/human CD11b, Clone Ml/70, Lot B296805, Cat 101226 (Biolegend)
- PE/CY7 anti-mouse F4/80, clone 8M8, Lot 8280917, CAT 123114 (Biolegend)
- APC anti-mouse LY6G clone 1A8, Cat 127614, Lot 8296099 (Biolegend)

Validation

- https://www.abcam.com/e-cadherin-antibody-m168-c-terminal-ab76055.html
- https://www.sigmaaldrich.com/DE/en/product/sigma/c7930FACS:
- https ://www. bd biosciences .com/en-de/ products/ reagents/fl ow-cytometry-reagents/ research-reagents/single-color -antibodies-ruo/ pe-rat-anti-mouse-cd8a.553032
- https ://www.thermofisher.com/antibody /product/IF N-ga m ma-Antibody-clone-XMGl-2-Monoclonal/17-7311-82
- https ://www.biolegend.com/en-us/products/pe-cyan lne 7-a nti-mouse-il-17a-anti body-6013 ?Groupl D=G ROU P24
- https://www.biolegend.com/en-us/products/apc-cyanine7-anti-mouse-human-cdllb-antibody-3930?Group1D=BLG10616
- ht tps ://www. bd biosciences. com/en-us/ products/reagents/fl ow-cytom etry-reagents/resea rch-reagen ts/ single-col or-an ti bodies-ruo/ pe--hamster-an i-mouse-cdllc.553802
- https ://www.biolegend.com/en-us/products/pe-cyan ine 7-a nti-mouse-f 4-80-antibody-4070 ?Grou pl D=BLG5319
- htt ps ://www. biol egen d .com/en-us/products/ a pc-anti-mou se-ly-6g-a nti body-6115 ?Group ID= BLG 72 34
- https ://www.biolegend.com/en-us/products/percp-cyani neS-5-a nti-mouse-cd3-anti body-55967GroupID= B LG 242
- https://www.bdbiosciences.com/en-de/products/reagents/flow-cytometry-reagents/research-reagents/single-color-antibodies-ruo/fitc-rat-anti-mouse-cd4.553046

Animals and other research organisms

Policy information about studies involving animals; ARRIVE guidelines recommended for reporting animal research, and Sex and Gender in Research

Laboratory animals	Epithelial intestinal cell-specific knock-out (Bmallfl/fl x Villin cre/wt) male mice and their control litter mates (Bmallfl/fl x Villin wt/wt) on a genetic C57BL/6J background (sacrificed at the age of 18-20 weeks) as well as germ-free wildtype male mice on C57BL/6J background (sacrificed at the age of 15-16 weeks) were used in this study. Both SPF and GF mice were single-housed in at 22 ± 1°C with a 12-h light/dark cycle unless otherwise specified in the manuscript.
Wild animals	no wild animals are used in this study
Reporting on sex	in our study we used male mice, since previous research show gender based differences in microbial rhythmicity.
Field-collected samples	no field-collected samples are used in this study
Ethics oversight	Experiments were conducted at Technical University of Munich in accordance with Bavarian Animal Care and Use Committee (TVA ROB-55.2Vet-2532.Ve _02-18-14),

Note that full information on the approval of the study protocol must also be provided in the manuscript.

Flow Cytometry

Plots

Confirm that:

- [x] The axis labels state the marker and fluorochrome used (e.g. CD4-FITC).
- [x] The axis scales are clearly visible. Include numbers along axes only for bottom left plot of group (a 'group' is an analysis of identical markers).
- [x] All plots are contour plots with outliers or pseudocolor plots.
- [x] A numerical value for number of cells or percentage (with statistics) is provided.

Methodology

Sample preparation	Immune cells were isolated from freshly isolated jejunum and colon. Intestinal tissues were flipped, washed out and cut into 1 cm pieces. To remove epithelial cells, pieces were incubated in DMEM with 20 µL of 1M DTT. After shaking for 15 minutes, tissues were incubated PBS with 200 µL of 150mM EDTA at 37°C with shaking. After 3 times harsh shaking in Hanks buffer, jejunum tissue was then digested at 37°C for approximately 15 min in Thermoshake (200rpm) with 0.6 mg/ml type VIII collagenase (Sigma-Aldrich). Colonic tissue was simultaneously digested under the same condition for approximately 25 min but with the mixture of 0.85 mg/ml type V collagenase (Sigma-Aldrich), 1.25 mg/ml collagenase D (Sigma-Aldrich), 10µl/ml Amphotericin (100x) 1 mg/mL Dispase II, and 30U/ml Deoxyribonuclease I (Sigma). Following digestion, intestinal cells were passed through a 40 µm strainer. Consequently, cells were fixed with 2% PFA, washed, and stored in RPMI at 4 °C until further processing.
Instrument	LSR-11 (SD Biosciences) flow cytometer
Software	FlowJo 10.7.2
Cell population abundance	All the samples were measured exact 2 minu es with the same speed to get minimum 20000 events for raw counts and percentage analysis.
Gating strategy	FSC A/SSC A was used to gate on the main cell population and remove debris. Followed by rsc A{rsc H gating to select singlet cells. Subsequent relevant gating was conducted, details can be checked in the gating strategy figure. All positive/ negative gating were defined based on FMO (Fluorescence Minus One) controls, and single staining histograms if needed.<br>All the samples were measured twice with different antibodies to avoid channel conflicts.

- [x] Tick this box to confirm that a figure exemplifying the gating strategy is provided in the Supplementary Information.
